# Complexity and data mining in dental research: A network medicine perspective on interceptive orthodontics

**DOI:** 10.1111/ocr.12520

**Published:** 2021-09-14

**Authors:** Tommaso Gili, Gabriele Di Carlo, Silvia Capuani, Pietro Auconi, Guido Caldarelli, Antonella Polimeni

**Affiliations:** ^1^ Networks Unit IMT School for Advanced Studies Lucca Lucca Italy; ^2^ CNR‐ISC Unità Sapienza Rome Italy; ^3^ Department of Oral and Maxillo‐Facial Sciences Sapienza University of Rome Rome Italy; ^4^ Private Practice Rome Rome Italy; ^5^ Department of Molecular Sciences and Nanosystems Ca’Foscari University of Venice Venezia Mestre Italy

**Keywords:** artificial intelligence, big data, complexity, machine learning, musculoskeletal magnetic resonance imaging, network medicine, orthodontics

## Abstract

Procedures and models of computerized data analysis are becoming researchers' and practitioners' thinking partners by transforming the reasoning underlying biomedicine. Complexity theory, Network analysis and Artificial Intelligence are already approaching this discipline, intending to provide support for patient's diagnosis, prognosis and treatments. At the same time, due to the sparsity, noisiness and time‐dependency of medical data, such procedures are raising many unprecedented problems related to the mismatch between the human mind's reasoning and the outputs of computational models. Thanks to these computational, non‐anthropocentric models, a patient's clinical situation can be elucidated in the orthodontic discipline, and the growth outcome can be approximated. However, to have confidence in these procedures, orthodontists should be warned of the related benefits and risks. Here we want to present how these innovative approaches can derive better patients' characterization, also offering a different point of view about patient's classification, prognosis and treatment.

## 1 INTRODUCTION

The technological revolution we have been experiencing in the last decades impacted medical sciences in various ways. Many results from high‐throughput experiments and the increased connection between data scientists have made available an unprecedented amount of insights to researchers and practitioners. Consequently, it becomes evident that many biological processes, as craniofacial growth, are prominent examples of complex systems in which the outcome depends upon the interaction of various components.^1^, ^2^ Each element depends on its past properties, the properties of the other elements, and their past. This new scientific paradigm triggered the search for mathematical instruments to describe and possibly drive such complex behaviour.^3^ A complex system of interacting agents takes a natural mathematical form of a graph where the vertices are the elements of the system, and their complex interaction is put in the form of an edge (directed, weighted, etc).^4^ By considering the individual variability within patients, network theory makes it possible to optimize and individualize the diagnosis in the modelling of precision medicine.^5^, ^6^ In the last decades, the evolution of disparate, complex biological systems has been predicted, almost automatically, by using different tools and procedures from Artificial Intelligence (AI).^7^, ^8^, ^9^, ^10^

From an initial data set of patient's characteristics (‘learning set’), AI algorithms learn how the features relate to and predict the outcome. Machine Learning (ML), a sub‐discipline in AI, is instead focussed on the ability to handle noisy or irrelevant data and on the capability to predict the outcome of a disease based on data derived from similar conditions.^11^, ^12^ The first attempts to use biomedical data to extract prediction resulted in probabilistic models trained on a series of case studies, tried to match the individual patient's condition with predefined classes of stratified increasing health risks.^13^

Using Artificial Neural Network procedures, the correlation between early craniofacial features and the risk of craniofacial worsening during growth was established in 43 orthodontic patients.^14^ As the number of cases available for predicting health outcomes increased, new problems related to data incompleteness and inhomogeneity and ethical aspects of storage arose.^15^, ^16^ Using Graph analysis, feature covariance across orthodontic patients allowed to disentangle Class III subjects from the topological patterns of other malocclusions.^17^ ML algorithms have been adopted to assist the orthodontist in treatment plan decision, including premolar extraction.^18^, ^19^ Indeed, such essential decisions during orthodontic treatments can be subjected to different points of view, as they tend to be based on the practitioner's experience and intuition. Resorting to ML procedures, one can decrease, at least in principle, personal biases in the treatment analysis and choices.^19^

The precision of ML answers crucially depends on the quality of data input, both in terms of number and appropriateness. ML algorithms cannot work if provided with raw data, which must be transformed into domain‐specific representative and salient information. Therefore, choosing the best characteristics (the most expressive of the problem) is crucial for predicting. It is also imperative to collect and give the machine data expressed as geometric, physiological, clinical and anamnestic parameters to make the most of what the current technology has to offer.^17^, ^20^, ^21^, ^22^

The integration of biomedicine and computer science is based on two concepts: Systems of the body have complex and dynamic biological properties that rely on the interaction of molecular agents sustaining the physiological functioning, as well as the pathogenesis of diseases at different scales; if complex interactions cannot be understood or processed by conventional methods, they can be investigated and explained using ML. The quantification of imaging biomarkers able to witness the status of a system of the body is the core business of medical research. Among the multiple choices available in the field, Magnetic Resonance Imaging (MRI)^23^ is the one that offers high accuracy and reliability at multiple spatial resolutions, with the invaluable plus to be non‐invasive.

This work aimed to present an essential overview of ML techniques and possible applications in the orthodontic discipline, highlighting merits, potentials and potential improvements offered by the inclusion of networked multiscale musculoskeletal data congruently segregated for advanced AI procedures. The integration of biomedical databases and mathematical models in multiscale and multi‐physics systems is a well‐established research field^24^, ^25^ that discusses dynamic and static mechanical dynamics as essential elements in constructing models of living organisms and diseases. Although it is well beyond the ultimate purpose of this paper, we feel confident that the inclusion of such modelling in the fusion of biomedicine and computer science presented here will be mandatory in the next future.

### 1.1 The orofacial system as a hierarchical multiscale complex system

Orthodontic researchers and practitioners are interested in extracting the most significant possible amount of information from all potential sources to provide the best diagnostic framing and treatment. It means mining significant patterns from past studies and tracing down the localization of the pathobiology underlying growing trajectories with and without unfavourable dentoskeletal growth. Such difference may point towards the prediction of modifiable skeletal disharmonies.^26^ However, it is problematic to understand a priori what would be the best data representation, and transform inputs into information that the machine could understand.

It is well recognized that bones and teeth structural and mechanical properties,^27^ and the elastic properties of muscles strongly influence the post‐treatment stability.^28^

**FIGURE 1:**
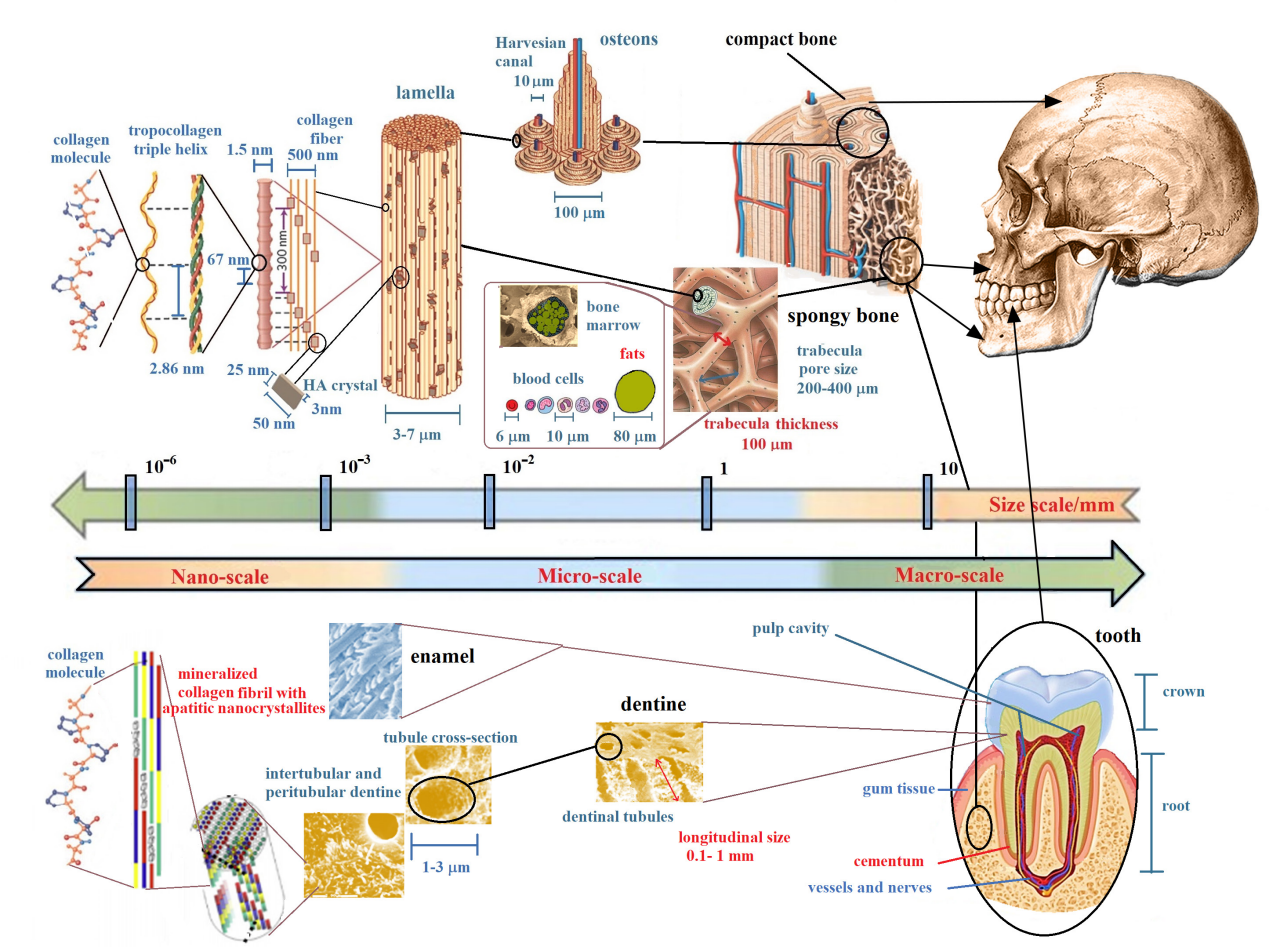
Schematic depiction of bone and tooth multiscale hierarchical structure from macroscopic bone (up raw) and tooth (bottom raw) to their nanoscopic elements. This hierarchical organization is the genesis of bone and dentine properties, including stress tolerance, adaptability and development during the growth process. In dentine, tubules are the prominent structural feature at a micro‐level, whereas collagen fibres decorated with apatite crystallite platelets dominate the nanoscale. In bone, hydroxyapatite (HA) crystals at nanometer‐level periodically are deposited within the gap zones of collagen fibrils during the bone biomineralization process. This hierarchical arrangement produces nanomechanical heterogeneities, which enable a mechanism for high energy dissipation and resistance to fracture. At a micro‐macro‐level, bone marrow quality in spongy bone and trabecular network rearrangement affects the resistance of bone to mechanical stress. Adapted from reference^24^, ^26^, ^65^, ^66^, ^67^

**FIGURE 2:**
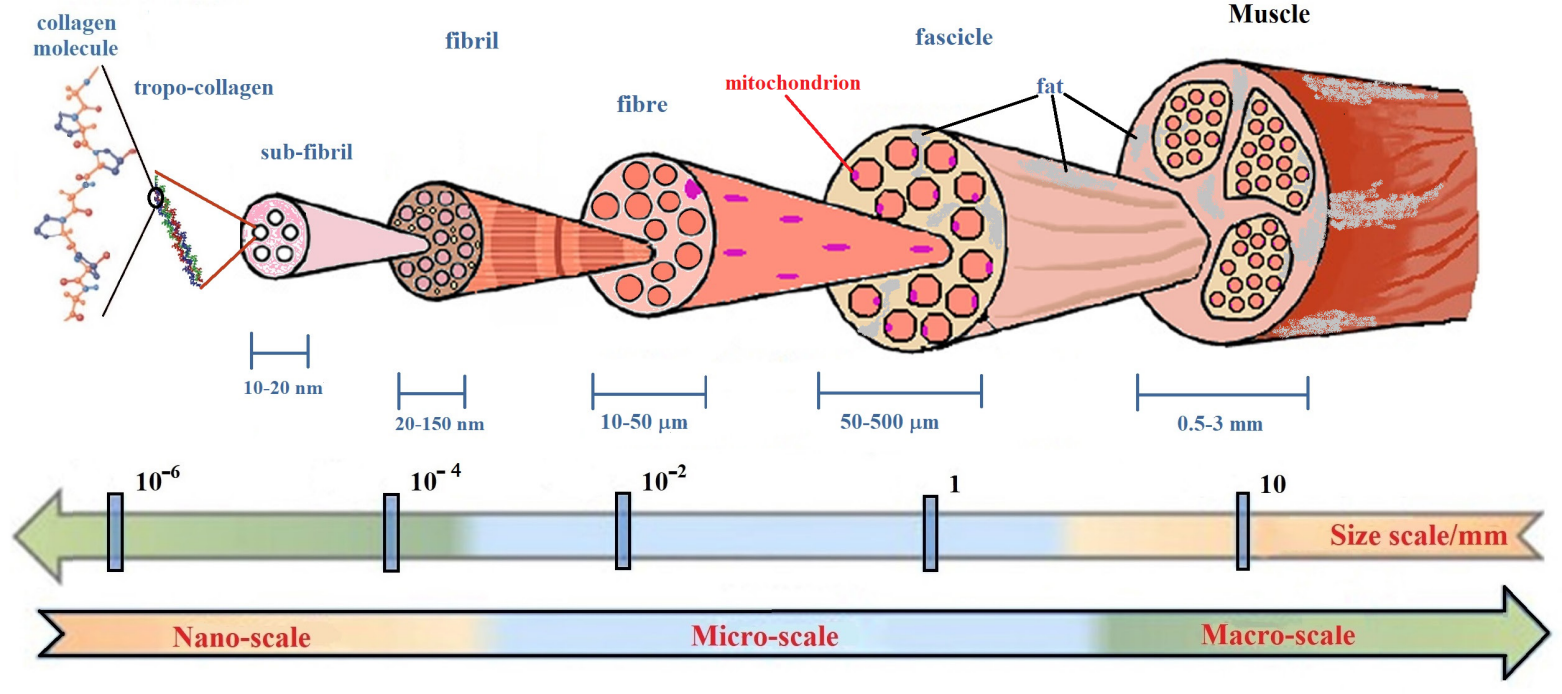
Schematic representation of muscle multiscale hierarchical structure. Most of the physiological muscle functions are related to its hierarchical organization and components. Physical inactivity causes a decrease in muscle mass and an increase in fat mass, but a chronic high fat diet also increases muscle fat, limiting full muscle function. Muscles quality is often related to skeletal tissue quality. Parameters that quantify the craniofacial muscles quality of an orthodontic patient should constitute the data set to be collected for planning treatment. Adapted from references^40^, ^41^

Once conceptualized as a whole, bones, teeth and muscles represent a complex system whose physiology is related to their multiscale hierarchical structures and the precise organization of inorganic and organic phases at the nanoscale, microscale, up to the macroscopic scale (Figures 1,2).

It is known how the effects of orthodontic treatments can lead to significant or poor results, depending on the period of growth during which it is implemented and on the quality of the bone and bone‐tooth interface.^29^ These latter factors involve both the mineral part and the collagen of bone marrow (Figure 1).^30^, ^31^, ^32^ Therefore, it is crucial to intervene with orthodontic treatments in the most appropriate period of growth but only to know the characteristics of the cancellous bones. Currently, there are attempts to evaluate both the optimal period of treatment and the health of the cancellous bone related to vertebral maturation. The maturation of the different skeletal segments does not happen at the same time. For example, the upper and lower jaw complete their development at other times.^33^ Due to the quality of the bone, the teeth can move or rotate unexpectedly, even with a technically perfect orthodontic procedure. Parameters that quantify the upper and lower jaw spongy bone quality should constitute the dataset to be collected for an appropriate and inclusive treatment plan. It is worth quantifying MRI parameters such as T2* and Apparent Diffusion Coefficient (ADC),^34^, ^35^, ^36^, ^37^, ^38^ which are related to spongy bone quality. Efforts to translate new dental and bone microstructural investigations to clinical practice in orthodontics should be devoted to making reliable predictions about the evolution of dentoskeletal tissue. It is crucial to understand how the micro and nanoscopic rearrangement contribute to the macroscopic bone tissue modification.^32^

Cells of the musculoskeletal system reside within complex and often interconnected 3D environments, which regulate musculoskeletal physiology and homeostasis (Figure 2). Maxillofacial development is also regulated by the surrounding soft tissues. Muscles affect skeletal components' size and shape with a possible different relationship between the muscular pressure and structural configuration in Class I, II and III malocclusions.^39^ Several orthodontic studies suggest the usefulness of monitoring electromyographic (EMG) activity of the jaw muscles. Moreover, a close correlation between skeletal and muscle tissues states has been recently highlighted,^40^ and fat increase and decreased muscle function. Parameters such as MRI T2 and ADC have proven useful to evaluate the muscles' state because of the relationship between the microscopic structure and function.^41^, ^42^, ^43^, ^44^

As highlighted above, despite the multiscale hierarchical nature of the musculoskeletal tissue, only macroscopic cephalometric variables composed of linear and angular geometric measurements are considered in conventional clinical practice. However, due to the development of in vivo imaging technology, additional methods can be used to obtain a set of multimodal and multi‐parametric measurements at different length scales, involving the microstructure, the topology of the craniofacial tissue, and also its physiology.^45^ In recent years, in addition to the conventional x‐ray computer tomography (CT) and the cone‐beam computer tomography (CBCT), MRI has shown great potential in dentistry.^46^, ^47^, ^48^, ^49^, ^50^ Furthermore, as most patients receive orthodontic treatment during childhood, further development of radiation‐free techniques, such as MRI are highly desirable.^51^, ^52^, ^53^

Together with the measurements provided by the imaging techniques, it is crucial to collect clinical and anamnestic data from patients. Some authors use genetic data to obtain a set of priors that could better supplement the inputs for correct patient classification.^54^ Modelling malocclusion progression means taking into account the complexity of the system. The complex interplay of causes behind atypical growth requires a different perspective about the disorders affecting the orofacial biological balance. By enlarging the basin of information about the system's chemical, physical and mechanical properties, it is possible to enable its description and evolution using more in‐depth ML and AI procedures. To achieve data more representative of the patient's situation, and therefore, the most significant possible amount of information, different in vitro investigation techniques about orofacial tissue at various scale lengths could be of clinical and research interest (Figure 2).^55^, ^56^ Once the most extensive possible spectrum of information has been obtained, deciding the best approach to combine the collected data are required. Here, we present different ML methods that stem from the complex network theory to offer the appropriate patients’ classification, without drawing on the choice of a specific and predictive biophysical model.

### 1.2 Learning features from data

**FIGURE 3:**
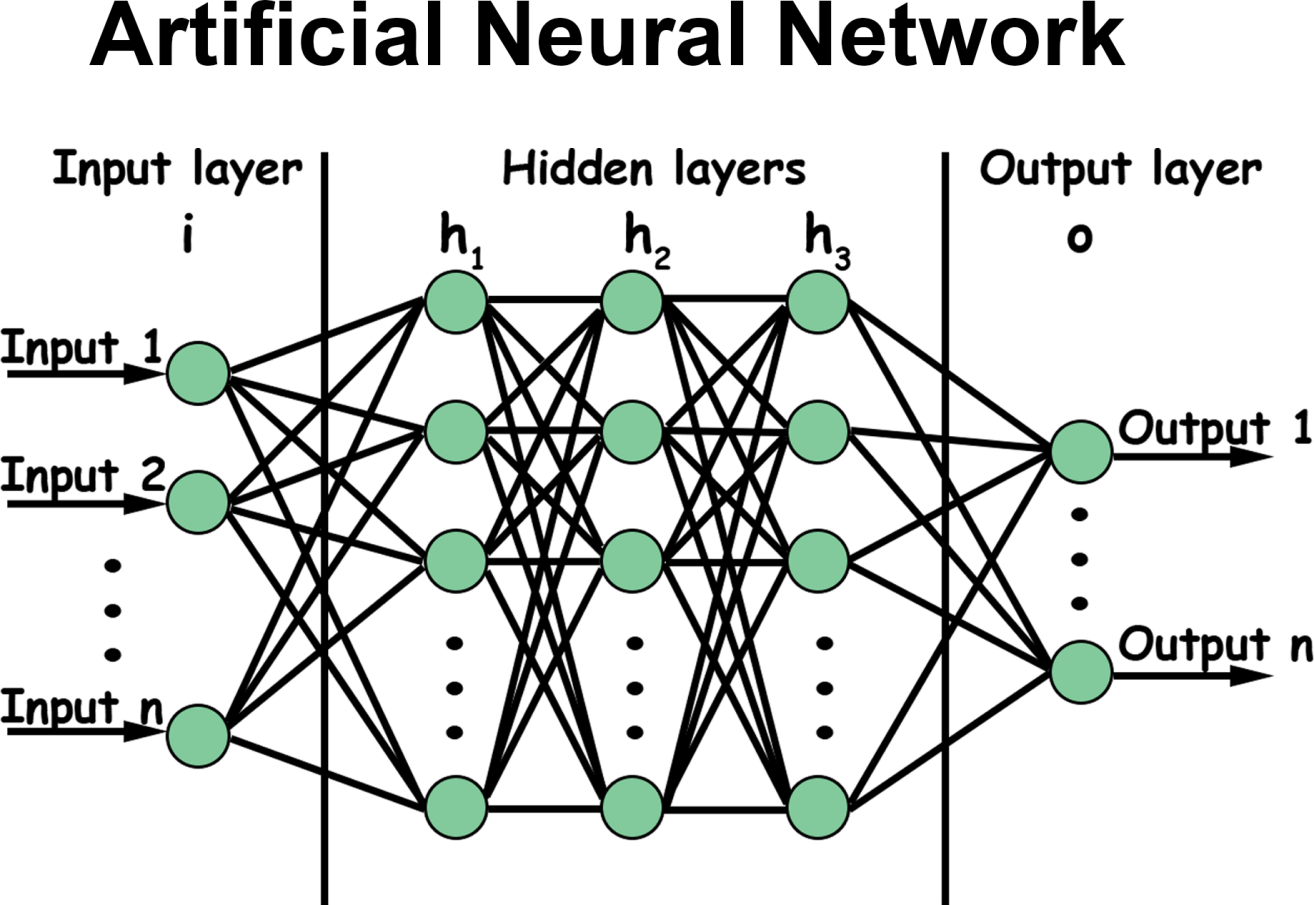
Artificial neural network schematization. An artificial neural network is based on a set of connected nodes, where connections, like synapses in a biological brain, can transmit a signal from a node to another. The transmitted signal is a real number, and the output of each node is computed by some non‐linear function of the sum of its inputs. Connections (also called edges or links) typically have a weight that adjusts as learning proceeds. Nodes are aggregated into layers, and different layers may perform various transformations on their inputs. Signals travel from the first layer to the last one (the output layer), often after going through the mid‐layers (hidden layers) multiple times. According to the number of layers included in the neural network, different machines can be realized, from simple machine‐learning engines to deep learning ones

Biomedical data, such as growth data, are constantly evolving. The related information is disseminated in a network of interconnected pieces of local information (nodes), and memory is encoded in the topology and the strength of such multiple connections (edges), rather than stored in the single information as in statistical databases. The power of such networks resides in their capacity to learn.^57^ Both in living beings and computer algorithms, the strengthening of connections is the microscopic mechanism for elaborating the information.^58^, ^59^ Artificial neural networks (ANN) use multiple layers of calculations to imitate the human brain's reasoning and draw conclusions from initial information. ANN can deal with complex intertwined problems (Figure 3). Different types of signs, symptoms, X‐rays, risk factors, imaging results enter into ANN algorithms to find the most predictive combination of variables. ANN assign weights to some combination of nodes (the features chosen to ‘train’ the machine) to repeatedly optimize the model's predictive performance.^60^ According to the number of layers included in the neural network, different machines can be realized, from simple machine‐learning (ML) engines to ‘deep’ learning ones.^61^, ^62^

The canonical ML workflow involves four steps: (a) data gathering, cleaning and pre‐processing, (b) feature extraction, (c) model training, (d) evaluation of results. Refining these steps can be complicated by intuition alone. The ability to separate patient's outcomes requires more targeted diagnostics, as to subgrouping patients with significantly different growth trajectories and clinical courses despite the similar early diagnostic frameworks.^60^, ^63^ Once ML approaches are matched to orthodontic data, challenges arise from data incompleteness, high‐dimensionality, heterogeneity, dynamicity, sparseness and statistical noise that can be partially mitigated by Network representation.^15^, ^64^ Due to such data's complex and interconnected nature, any single model can deepen only a tiny part of the entire orthodontic domain.^65^, ^66^ Whilst the conventional medical approach is based on the careful recruitment of clinical and laboratory data, testing of a diagnostic hypothesis, causes and effects of phenomena, significance, checking the initial hypothesis and so on, ML workflow focuses instead on the fast predictive performance of models and iterative improvement of the algorithms, coping with high‐dimensional spaces, variability of features and formats.^60^, ^67^

#### 1.2.1 Data gathering and Feature transformation

**FIGURE 4:**
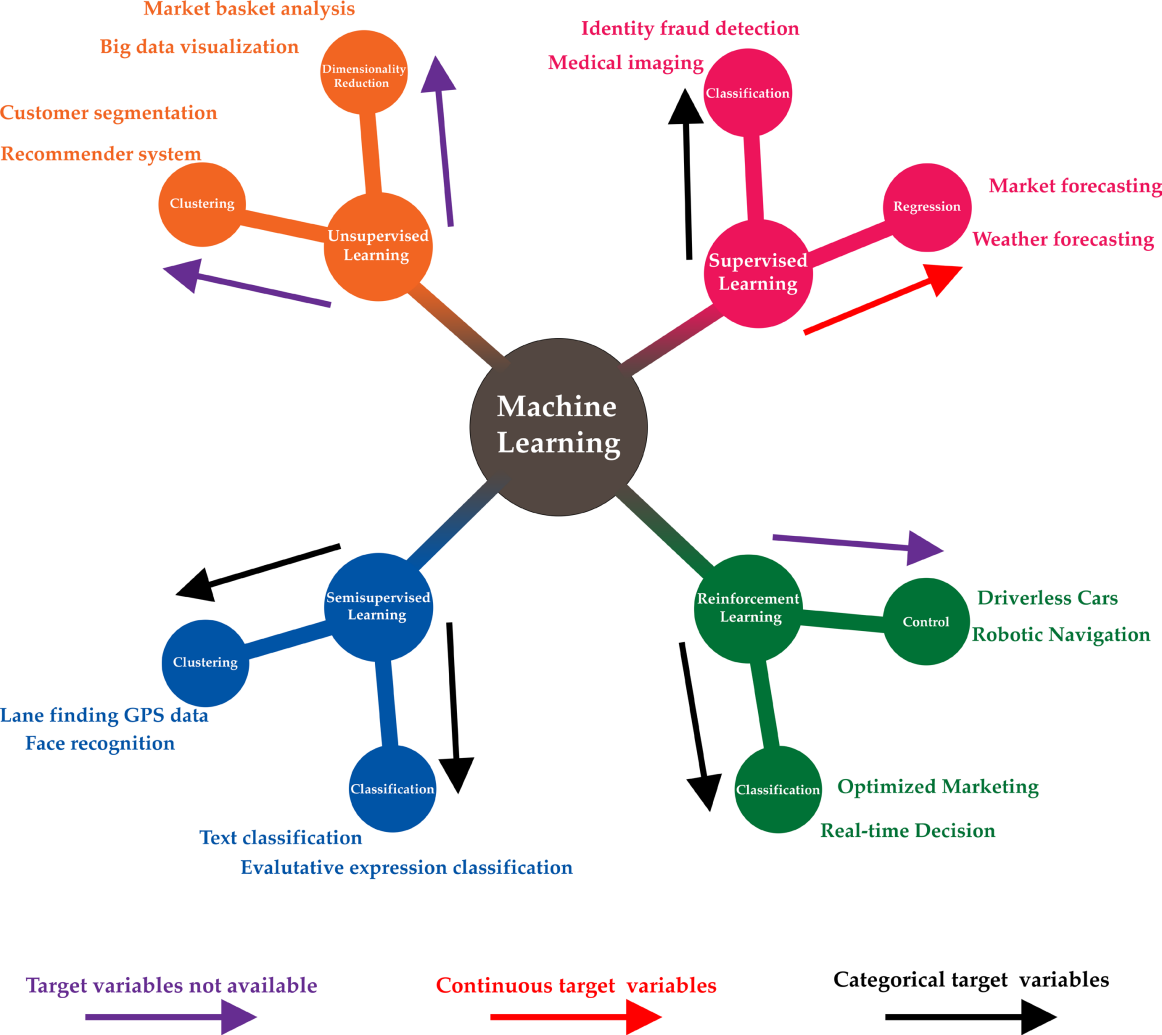
Machine learning. Different approaches to the learning process produce different machine‐learning schemes. According to the number of features used to train the machine, one can obtain supervised learning (labelled data sets are used to train the machine), unsupervised learning (unlabelled data sets are used to train the machine), semi‐supervised learning (a mix of labelled and unlabelled data sets are used to train the machine) and reinforcement learning (there are not data sets to train the machine). According to the quality of the target data (continuous or categorical), different tasks can be performed by the learning processes

Feature engineering (FE) is the process that transforms raw data into features to feed into the prediction models. Therefore, features sit between data and models in the ML workflow (Figure 4). Moreover, being interested in defining the quality of growth, in the beginning, it has to decide how to assign each patient to the proper category: as an example, favourable/unfavourable growth often represents the label of interest. Then, patients' clinical and cephalometric data can be imported into the training data, together with the specific labels. It is the beginning of the supervised learning approach. The algorithm will find the function that links the patient's input data with the outcome, minimizing the number of errors. However, raw data can rarely be entered without a preliminary selection to discard redundancies and simplify the system.^68^

#### 1.2.2 Learning

Regularization is the crucial attribute for high‐quality learning data from a large number of features. The process does not aim to predict labels within data of the learning dataset optimally but rather generalize the prevision about new, previously unseen patients. Accordingly, data regularization is equivalent to imposing a penalty for the system's complexity to improve performance.^69^ When the algorithm has few patient samples available from which it can learn the connection between the characteristics and the outcome, it ends up memorizing the training data set instead of learning general features of data (over‐adaptation to data, a.k.a. ‘overfitting’). For this reason, a model will perform well on the training set and poor with new patient's data. On the other hand, a small number of patient's characteristics in the learning set provides an inadequate description of the problem at hand and may result in learning difficulties (‘underfitting’). Thus, there is a trade‐off between the model performance and the size of the training set, that is, the patient coverage, the per cent of potentially eligible patients for that risk assessment can be completed.^69^

Supervised ML learning is used when the output is known. The learning procedure primarily deals with regression and classification problems. It focuses on classification, which involves choosing between subgroups to describe a new patient best, and predictions, which consists of estimating an outcome of interest, such as the quality of future craniofacial growth.^63^, ^69^

In contrast, unsupervised learning is not intended to find outputs to predict, and it tries to find naturally occurring patterns within data.^10^ The patterns identified have to be evaluated for utility. Unsupervised models aim to discover groupings from data samples ‘x’ without knowing the label output ‘y’. The algorithm is provided with unclassified data records to recognize whether any existing latent patterns are present. Clustering, dimensionality reduction, like principal component analysis are leading examples of unsupervised learning approaches. The preliminary subdivision into subgroups of patients may lead to the subsequent estimation of specific risk factors. In particular, clustering refers to the extraction of group latent similarities within data that allow subjects to be grouped into subsections. The clustering assumption states that such subsections of subjects often exhibit the same outcome.^70^ In orthodontics, because of the complexity of medical data and heterogeneity of patients, identifying subsections of patients by intuition can be difficult.^20^

Semi‐supervised learning (SSL) is a mixture of supervised and unsupervised models. It analyses several unlabelled cases (patients) whilst augmenting its pattern recognition capacities with a small quantity of labelled data.^71^

This approach is promising in orthodontic longitudinal studies since it is laborious to find data that refer to many patients followed closely over time, at comparable regular intervals; even more, untreated patients followed longitudinally.

Finally, reinforcement learning (RL) is an approach where software agents take actions in an environment to maximize a cumulative reward. RL differs from SL in not needing labelled input/output pairs to be presented without requiring sub‐optimal actions to be corrected. The goal is finding a balance between exploration (of uncharted territory) and exploitation (of current knowledge).^72^, ^73^

### 1.3 Learning features from networks

**FIGURE 5:**
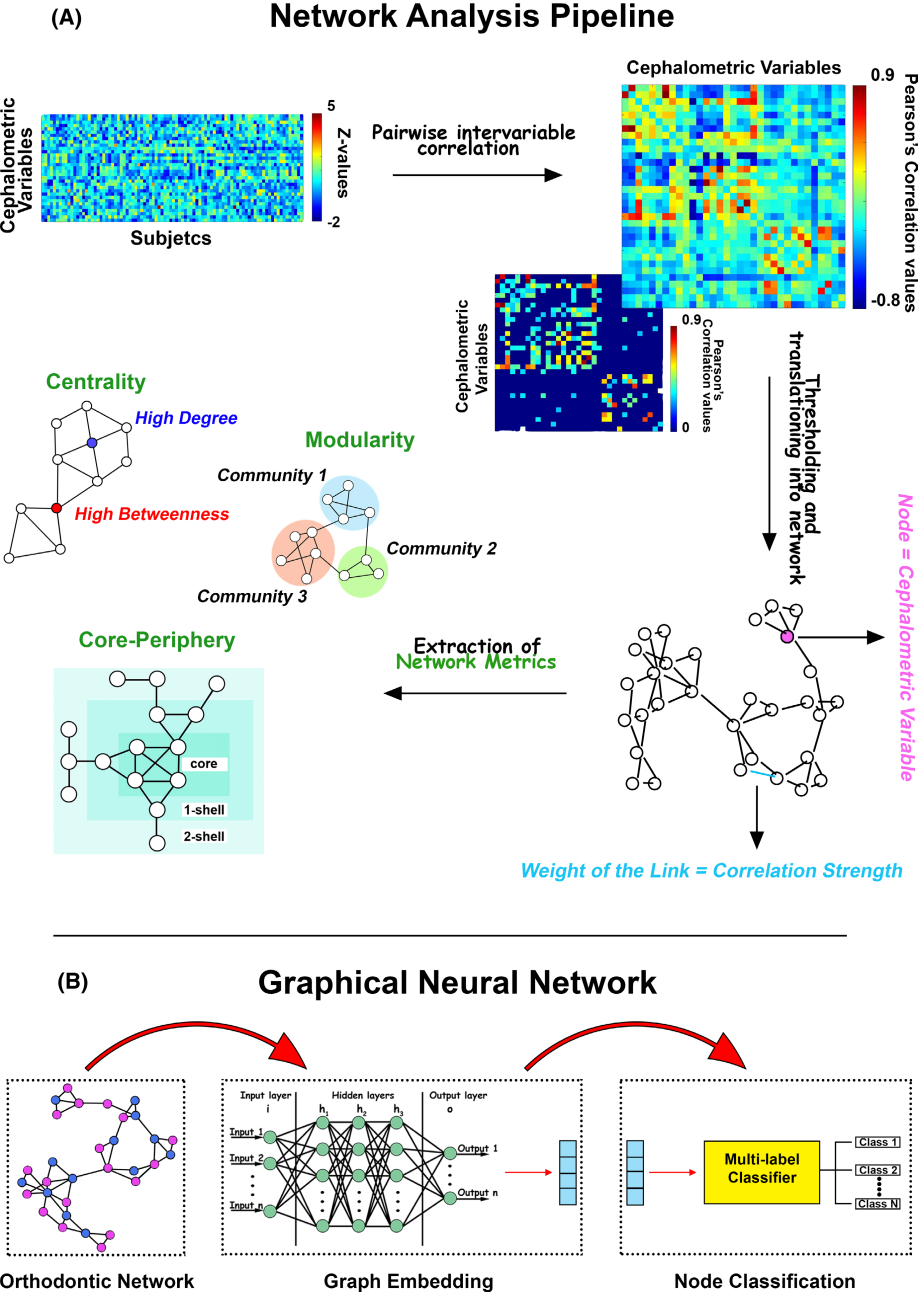
Complex Networks and data analytics. (A) Network analysis pipeline for orthodontics data. Once cephalometric variables are standardized to Z‐values, they are entered in a cross‐correlation process that returns a symmetric matrix, whose entries are the intervariable Pearson's correlation coefficients across subjects. A threshold is set to the matrix according to the *P*‐values associated with the coefficients. The final matrix (a weighted adjacency matrix) is translated into a network whose nodes are the cephalometric variables and the weights of the links the Pearson's correlation coefficients that survived the thresholding process. Finally, different metrics have been calculated from the network topology: centrality measures, modules or communities and the core‐periphery structure. (B) GNN. Low‐dimensional node representations are first learned from networks by graph embedding and then used as features to build specific classifiers for different tasks

Learning techniques rely on networked environments to conduct the learning process on data of interest. What if data are not generated from grid‐like Euclidean structures (like images and videos) and represented as graphs with complex interdependencies between objects? For network‐based methods, the learning procedure is performed by navigating in networks built from the input data according to some similarity criterion.^74^ As networks naturally contain topological information of data relationship, network‐based methods take advantage of typical algorithms that use raw data. It must be stressed that network‐based methods can be considered a general solution for learning tasks, even for data sets not represented by networks. One can apply network construction techniques to that data set to generate a network from the input data. Once the network is constructed, the learning process can be run. Patient stratification is a general target of network‐based methods of machine learning. For example, cancer somatic mutation profiles are highly coupled with the biomolecular network,^5^, ^75^ in fact, somatic mutations of a cancer driver gene may lead cancer genome evolution to mutations in other genes.^76^ Therefore, each patient with its somatic mutation profiles can be identified, and the similarity between patients can be used to mine for tumour stratification. Analogously, information about malocclusion within the orofacial system can be extrapolated from the correlation matrix of the orthodontic features. The correlation matrix relates to a network whose vertices are associated with the features and the edges to their covariance across patients,^17^ allowing the visualization of malocclusion information (Figure 5A). The networks are obtained by fixing a threshold value T: if the Pearson correlation between two vertices (features) is larger than T, they are considered connected (“linked”).^17^ Several topological network metrics are typically considered to convey biological meaning to cephalometric correlations (Figure 5A), as ‘betweenness’^20^ (a node centrality index) or modularity (the capacity of nodes to form communities characterized by similar properties).^77^ Once the graph is computed, the topological structure of the input is encoded in simpler structures (eg vectors of reals), and then the ML algorithms can be run.

The application of ML to graphs can be generally divided into two categories^78^:

• Node‐focussed applications, where the final task is associated with specific properties of each node (examples include node clustering, link prediction, semi‐supervised node classification).

• Graph‐focussed application, where the model to be realized is dependent on the whole graph structure (examples include estimation of properties of graphs and graph classification).

The machine‐learning methods that operate on graph domain are known as Graph Neural Networks (GNNs). Due to its convincing performance, GNN has become widely applied to infer data described by graphs.^78^, ^79^, ^80^ In GNN, the concept of Node Embedding is implemented. It means to map nodes to a d‐dimensional embedding space (low dimensional space rather than the actual dimension of the graph) to embed close to each other similar nodes in the graph. The procedure aims to map nodes, so the similarity in the embedding space approximates similarity in the network.^81^, ^82^ Training can be unsupervised, semi‐supervised or supervised. The supervised setting provides labelled data for training and is used for node classification. The semi‐supervised setting gives a small number of labelled nodes and many unlabelled nodes for training. In the test phase, the transductive setting requires the model to predict the labels of the given unlabelled nodes, whilst the inductive setting provides new unlabelled nodes from the same distribution to infer. Most node and edge classification tasks are semi‐supervised. The unsupervised setting only offers unlabelled data for the model to find patterns. Node clustering (features/patients grouping) is a typical unsupervised learning task.

### 1.4 Perspectives

The parameters necessary to understand and predict the evolution of an orthodontic condition (such as a malocclusion) cannot be captured by a single measurement modality or using a few data type (as an example, geometrical data from cephalometrics, from CBCT or anamnestic data). The dental‐musculoskeletal tissue complexity lies in the interconnection of morphological progression, functional and genetic data. Nowadays, new and more sophisticated AI algorithms can deepen these aspects. Unfortunately, they do not give a clue about the reasoning that led to deciding the saliency, the relevance or the causal importance of the variables.^83^, ^84^ Interestingly, theoretical advancements with methods from statistical physics will also allow determining partial information as estimating longitudinal data from cross‐sectional to create individual prognosis.^70^ Humans are brilliant at clinical pattern recognition in dimensions equal to or less than three. However, most medical data's dimension is much higher than three, making cognitive analysis problematic or even impossible.^6^

Multiple perspectives are required to study complex phenomena formed of parts in a non‐random organization, in which our knowledge can only be partial and idealized. Forcing biological explanatory pluralism into the narrow computational framework appears to be a misleading strategy. The complexity of the musculoskeletal craniofacial system requires the use of novel sources of information. In vivo, MRI matches this framework optimally, providing the elucidation of geometrical and physiological tissue features, even at length scales smaller than voxel resolution of X‐ray CT and CBCT in a radiation‐free modality. The computational models applied to orthodontics must include the skeletal characteristics change over time (even at the end of growth) and the relationship between the mechanical properties of the complex system composed of bones and teeth and their multiscale hierarchical structures, whose precise organization orchestrates the whole physiology. The question is to what extent this new ‘deep’ reasoning leads to reliable and responsible decisions in the global growth of the skeletal bases and the maturation of the dentition. The course of events almost always deviates from what was predicted and planned. There may be areas of local densification of skeletal disharmonies that can act as growth attractors. These areas are difficult to detect clinically and neither be easily detected by computational systems.

The future of orthodontics inexorably will pass through a Copernican revolution that will lead algorithms to optimize therapies (‘personalized medicine’). Nonetheless, the process will not be complete until the complexity of the problem is fully addressed. The need for new imaging techniques (in vivo and in vitro) to characterize teeth, bones and muscles structures and physiology, the availability of genetic data, and the filtration of helpful information from them will be the route to a new vision of orthodontics.

## CONFLICT OF INTEREST

The authors have no conflict of interests to declare.

## AUTHOR CONTRIBUTION

Conceptualization, TG, GDC; methodology, PA, SC.; writing—original draft preparation, TG, GDC; writing—review and editing, GC, AP; supervision, GC.

## Data Availability

Data sharing not applicable to this article as no data sets were generated or analysed during the current study.
